# Development-on-chip: *in vitro* neural tube patterning with a microfluidic device

**DOI:** 10.1242/dev.126847

**Published:** 2016-06-01

**Authors:** Christopher J. Demers, Prabakaran Soundararajan, Phaneendra Chennampally, Gregory A. Cox, James Briscoe, Scott D. Collins, Rosemary L. Smith

**Affiliations:** 1 Microinstruments and Systems Laboratory, University of Maine, Orono, ME 04469, USA; 2 Graduate School of Biomedical Sciences and Engineering, University of Maine, Orono, ME 04469, USA; 3 The Francis Crick Institute, Mill Hill Laboratory, London NW7 1AA, UK; 4 Moffitt Cancer Center, Tampa, FL 33612, USA; 5 The Jackson Laboratory, Bar Harbor, ME 04609, USA

**Keywords:** Microfluidic, Differentiation, Stem cell, Neuron, Patterning, Mouse

## Abstract

Embryogenesis is a highly regulated process in which the precise spatial and temporal release of soluble cues directs differentiation of multipotent stem cells into discrete populations of specialized adult cell types. In the spinal cord, neural progenitor cells are directed to differentiate into adult neurons through the action of mediators released from nearby organizing centers, such as the floor plate and paraxial mesoderm. These signals combine to create spatiotemporal diffusional landscapes that precisely regulate the development of the central nervous system (CNS). Currently, *in vivo* and *ex vivo* studies of these signaling factors present some inherent ambiguity. *In vitro* methods are preferred for their enhanced experimental clarity but often lack the technical sophistication required for biological realism. In this article, we present a versatile microfluidic platform capable of mimicking the spatial and temporal chemical environments found *in vivo* during neural tube development. Simultaneous opposing and/or orthogonal gradients of developmental morphogens can be maintained, resulting in neural tube patterning analogous to that observed *in vivo*.

## Abstract (precis)

**Summary:** A microfluidic device mimics the spatial and temporal environment of neural tube development *in vivo* and enables the correct spatial organization of neural tube formation from stem cells *in vitro*.

## INTRODUCTION

**Fig. 1.**
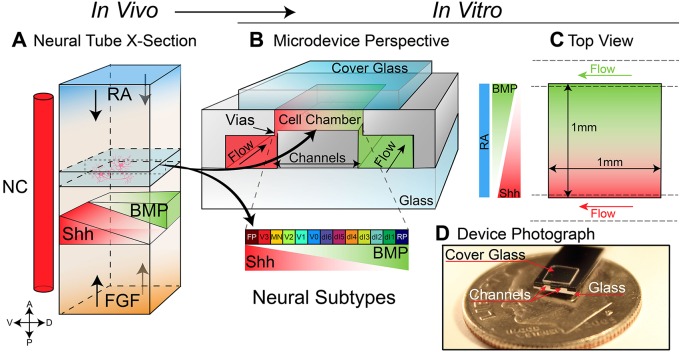
**Graphical overview of the microfluidic reconstruction of the neural tube.** (A,B) Schematic of a neural tube highlighting the 100 µm ‘slice’ recreated by the microfluidic device. Four primary signals (RA, SHH, BMP and FGF) are responsible for patterning the bulk of the neural tube (NC, notochord) (A). The SHH gradient, which is responsible for directing the differentiation of ventral neural progenitors into discrete domains of neurons, is recreated inside the cell culture chamber of the microdevice (B). Flow channels running under the cell chamber supply nutrients as well as desired guidance molecules to the cells in the culture chamber. Morphogen concentration gradients are established across the chamber using the vias in a standard source/sink configuration with the walls of the chamber acting as reflective boundaries. (C) A top (rotated) view of B, as seen through the cover glass. (D) A photograph of the microdevice sitting on top of a dime indicates the scale of the device. FP, floor plate; RP, roof plate.

During spinal cord development, organizing centers surrounding the neural tube, such as the notochord, paraxial mesoderm and roof/floor plates, release chemical cues directing neural precursor cells to differentiate into mature neurons (Fig. 1). The most studied of these cues is sonic hedgehog (SHH), which is generated in notochord and floor plate cells establishing a ventral (high) to dorsal (low) concentration gradient across the neural tube (Ribes and Briscoe, 2009) directing the differentiation of ventral neural progenitors (Roelink et al., 1995) into highly organized domains of neural subtypes (Bushati and Briscoe, 1994). An opposing gradient of bone morphogenetic protein (BMP) and other members of the transforming growth factor beta (TGFβ) superfamily of signaling molecules are simultaneously released by roof plate cells, concurrently patterning the dorsal half of the neural tube and establishing a cross-repressive boundary between the dorsal and ventral halves of the developing spinal cord (Le Dréau et al., 2012). Patterning along the anteroposterior (AP) axis occurs simultaneously with dorsoventral (DV) patterning and is the result of opposing gradients of retinoic acid (RA) and fibroblast growth factor (FGF)/WNT, which induce a sequential activation of homeobox (Hox) genes (Diez del Corral et al., 2003; Liu et al., 2001). Current models indicate that these four signaling molecules (SHH, BMP, RA and FGF) jointly coordinate most of the spatial and temporal differentiation of the neural tube (Wilson and Maden, 2005).

Much of the detail of embryonic patterning, such as the interaction between signaling pathways, remains unknown (Wilson and Maden, 2005). To this end, the directed differentiation of stem cells to recapitulate developmental events provides an experimental approach to shed light on these complex developmental processes (Gouti et al., 2014; Turner et al., 2014; van den Brink et al., 2014). However, without appropriate tools to exert spatial control over the growth and differentiation of stem cells, researchers are limited to simple bath applications of differentiation factors.

To preserve developmental organization *in vitro*, we have designed, fabricated and demonstrated a microfluidic device capable of recreating the tightly regulated microenvironment of chemical morphogens found within developing tissue. The microdevice employs simple Fickian diffusion (Smith et al., 2010) principles to generate concentration landscapes of crucial morphogens that faithfully mimic the *in vivo* environment responsible for directing naïve stem cells to differentiate into their specialized cell destinies. To date, only two other microfluidic platforms have been used to study aspects of spinal cord patterning (Amadi et al., 2010; Park et al., 2009); however, neither were able to recreate the spatially discrete domains of neural subtypes found in the neural tube. As a result, to the best of our knowledge, this is the first report of the recapitulation of the spatial organization of neural tube development *in vitro*.

## RESULTS

### Microdevice design, fabrication and characterization

Culturing stem cells in sub-microliter volumes adds a complexity not found in typical macro-scale culture. Small perturbations, such as changes in pH, spurious signaling factors or metabolites, can lead to significant changes in the local microenvironment. As a result, we fabricated our microdevice entirely in silicon and glass, which are both biocompatible and bioinert. This avoids the chemical absorption and leaching problems introduced by polydimethylsiloxane and other polymer-based devices (Regehr et al., 2009; Łopacińska et al., 2013).

The overall geometry of the microdevice is designed to mimic the primary aspects of the diffusion-based patterning of the neural tube. The cell culture chamber (Fig. 1B) is 1 mm×1 mm×100 µm, which is similar in size to a thin (100 µm) slice of the developing chick or mouse neural tube (Smith and Schoenwolf, 1997). Microfluidic channels running beneath the cell chamber supply cells with nutrients and simultaneously remove waste products through small openings (vias) that connect the supply channels to the cell chamber. The chamber and vias are filled with Matrigel or Geltrex to provide a 3D culturing matrix for the cells. The matrix has the additional benefit that it also provides a viscous hydrodynamic barrier to fluid flow across the chamber, ensuring that the chamber chemical composition is defined entirely by diffusion. The presence of the matrix does not inhibit the diffusion of small molecules (i.e. glucose, differentiation factors, etc.), and even for large proteins, such as serum albumin, the diffusion coefficient has been shown to be identical for water and Matrigel (Shin et al., 2013). Moreover, the matrix can be remodeled by the cells and has been stripped of endogenous growth factors, thus providing a physiologically relevant substrate for growth and differentiation. A photograph of a fractured microdevice sitting on a dime is shown in Fig. 1D, revealing the microfluidic channels along the edge of the device. The cell culture chamber is covered with a thin glass cover slip (≈3 mm×3 mm×0.17 mm).

**Fig. 2.**
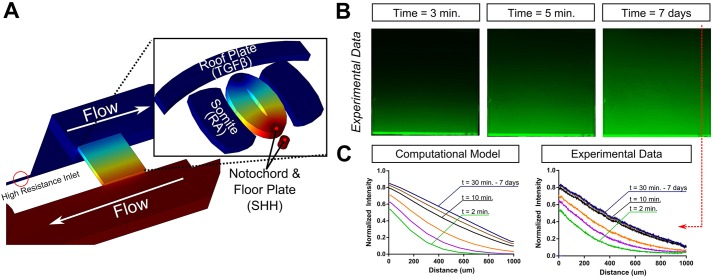
**Computer simulations of microdevice operation accurately predict device behavior.** (A) Using the actual microdevice geometry, a simple linear gradient is modeled in COMSOL, analogous to the SHH gradient established in the neural tube (inset). (B) Time-lapse imaging of the evolution of a fluorescein gradient inside the cell culture chamber of the microdevice, demonstrating that a concentration gradient can be established in the microdevice. (C) Quantification of fluorescence intensity in the predicted computational model and in the fluorescein gradient in the microdevice, highlighting the predictability of microdevice operation as well as the ability of the microdevice to maintain a stable linear gradient over 7 days. Quantification of images performed as one-dimensional average intensities as a function of distance across the microdevice. Quantification is repeated for several time points (2, 3, 5, 10, 15 and 30 minutes) to obtain temporal data.

To optimize the design and operation of the microdevice, we employed modeling software (COMSOL) to elucidate diffusion profiles within the cell chamber (Fig. 2). The physical laws governing the operation of the microdevice are Fick's laws of diffusion and the Navier–Stokes equations (Smith et al., 2010). The judicious placement of sources and sinks allows a wide variety of diffusion profiles to be engineered in the microdevice. Initially, a simple two-port system was used that is suitable for generating a linear concentration gradient. This gradient within the chamber corresponds with SHH diffusion profiles found in the dorsoventral axis of the neural tube (Fig. 2A, inset) (Chamberlain et al., 2008). For the factors employed in this study, linear concentration gradients can be established in <30 min and are maintained indefinitely, provided flow in the microchannels maintains boundary concentrations at the vias. The time to reach steady state is a function of both the diffusion coefficient of the diffusing molecule and the distance between source and sink as dictated by Fick's laws of diffusion (Smith et al., 2010). COMSOL calculations indicate that microchannel flows >1 µl/h are sufficient to maintain stable gradients across the microchamber. For all experiments reported here, we used 100 µl/h flow rates to guarantee fixed boundary conditions. In order to experimentally verify that a linear gradient is established and maintained, one channel of the device was supplied with fluorescein (source) whereas the other channel contained no fluorescein (sink). Fig. 2B shows images for the experimental development of the fluorescein gradient with time. Fluorescein provides a useful approximation for the diffusion dynamics of the small molecules used for motor neuron (MN) differentiation because of the similar size (520 Da and 332 Da for purmorphamine and fluorescein, respectively) and diffusion coefficients, which are 5.4×10^−6^ cm^2^/s for fluorescein and 2.4×10^−6^ cm^2^/s for RA (Dodson et al., 2002). The two graphs (Fig. 2C) plot the computer simulated (left) and experimental (right) fluorescein spatial concentration profiles across the device for a series of arbitrary times. The image and plots show that a linear gradient is maintained for the full 7 days of testing. At the completion of each experiment, device functionality was verified by introducing a dye, typically rhodamine or fluorescein, into one channel and verifying a gradient across the cell culture chamber.

### Directed spatial MN differentiation in the microfluidic device

Exerting spatial control over the differentiation of embryonic stem cells (ESCs) represents a significant step towards validating the microdevice as a viable platform. Therefore, we first used a modification of an established protocol known to induce motor neuron differentiation *in vitro* (Wichterle et al., 2002) to demonstrate that ESCs in the microdevice respond appropriately to chemical morphogens. ESCs containing an *HB9::*GFP transgenic reporter gene were suspended in a 3D gel matrix and seeded in the microdevice growth chamber, capped with a cover slip and supplied a uniform concentration of 1 µM RA and 3 µM purmorphamine (PM), a known agonist of the SHH pathway (Sinha and Chen, 2006), in ADFNK media (see Materials and Methods) for 7 days. *HB9* (*Mnx1* – Mouse Genome Informatics) is a homeobox gene only expressed by post-mitotic MNs (Arber et al., 1999), and is thus a useful marker for differentiation.

**Fig. 3.**
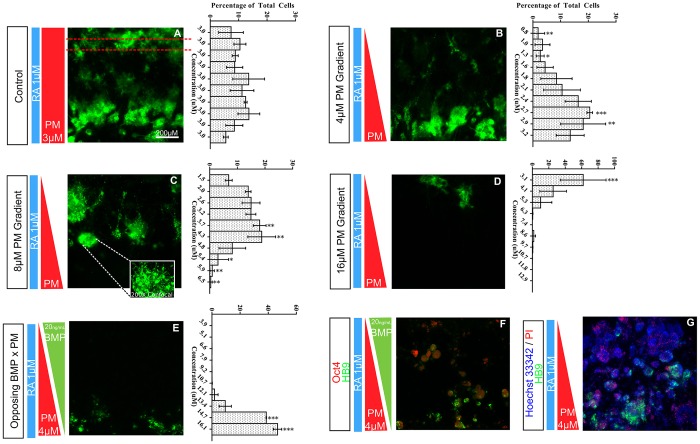
**Directed spatial patterning in the microfluidic device reveals a region of permissive differentiation.** Representative images and average plots of spatial differentiation of HB9^+^ cells (GFP labeled) along SHH gradient (*n*=4). Vertical bars on the left diagrammatically indicate the concentration and spatial gradient of RA and/or SHH. Plots to the right indicate the average intensity distribution from at least four experiments as well as actual PM concentrations based on computer simulations (quantified as mean percent cells/bin ±s.d., *n*=3). (A) Control HB9^+^ MNs subjected to a uniform concentration of PM and RA. Red dashed lines indicate example bin width. (B-D) HB9^+^ MNs subjected to varying PM gradients. Inset in C illustrates higher magnification detail of the MN cluster (200× confocal image). (E) The addition of an opposing gradient of BMP4 (20 ng/ml) further narrows the MN domain. (F) High expression of the pluripotency marker OCT4 towards the dorsal end of the microdevice (outside of the permissive MN region) indicates the effect of early exposure to a cross-gradient of BMP4. (G) Live/dead staining with Hoechst 33342 and propidium iodide (PI) reveal that we have not simply created a zone of permissive cell growth. *P≤0.05, **P≤0.01, ***P≤0.001.

After 7 days, cells were imaged for GFP expression in order to identify spatial patterning within the cell chamber (Fig. 3). Except where noted, all images were taken under low magnification (50×) to capture the entire 1 mm×1 mm cell chamber and fluorescence intensity was quantified as a function of spatial distribution down the SHH/PM gradient. For analysis, the chamber was divided vertically (along the gradient) into ten 100-µm-wide bins, and the fluorescence intensity, which is proportional to the number of *HB9*^+^ cells, in each bin quantified. All experiments were repeated on at least four different devices, and the average cell counts for all experiments plotted as the percentage of total cells to the right of the figure as a function of distance (additional details can be found in Materials and Methods).

After exposure to a uniform concentration of PM and RA, a uniform concentration of *HB9*^+^ cells populate the device, clearly validating the microdevice as capable of promoting ESC differentiation to MNs (Fig. 3A). We next exposed *HB9::*GFP ESCs to a linear gradient of PM in a uniform background of RA (Fig. 3B) from day 0 to day 7 to demonstrate the hypothesis that a SHH gradient is responsible for the stepwise remodeling of neural precursors into distinct subtypes of ventral neurons (Ericson et al., 1997). The background RA serves to drive neural differentiation and indeed is required for MN development both *in vitro* and *in vivo* (Novitch et al., 2003). In all figures, gradients are schematically represented as triangles and concentrations span from the indicated value (i.e. 4, 8 or 16) at the high end to zero at the opposite end. Interestingly, an *HB9*^+^ band of differentiated MNs resembling that found *in vivo* clearly developed. Quantification of replicate microdevice experiments (*n*≥4) using ANOVA confirmed a statistically significant non-uniformity in MN differentiation.

Previous work using chick neural tube explants has revealed that the time/dose-integral of the SHH gradient specifies ventral neural subtypes within the neural tube (Dessaud et al., 2010). To test this finding, we varied the PM concentration gradient and looked for corresponding shifts in the *HB9*^+^ band (Fig. 3B-D). Our results indicate that MN differentiation is strictly concentration driven. PM concentration profiles (Fig. 3, vertical axis on individual histograms) using calculated values from COMSOL simulations illustrates that MNs in the microdevice prefer an absolute PM concentration of ∼3-4 µM independent of the PM gradient magnitude or time to differentiation. This suggests that ESCs respond to neither the presence of a SHH gradient per se nor the slope of the SHH gradient, but rather to a narrow range of PM concentrations. This finding correlates well with *in vivo* results indicating that adjusting the diffusivity of the SHH ligand leads to spatial changes in MN development (Dessaud et al., 2008). Higher magnification (200×) confocal imaging revealed hundreds of neurons within each cluster (Fig. 3C, inset) and staining of microdevices *in situ* with Hoechst 33342 and propidium iodide (PI) confirmed that the vast majority of cells in the microdevice were living and we had not simply created a hospitable growth zone (Fig. 3G). Taken together, these results suggest that ESC differentiation can be spatially patterned by using a PM gradient to establish a permissive differentiation region.

The remarkable similarity between our *in vitro* results and known *in vivo* patterning prompted us to extend our study to include two opposing gradients, PM and BMP4, in order to explore additional controls that we could potentially exert over spatial differentiation. During vertebrate neural tube patterning, the signaling factor BMP4 mediates the differentiation of a subset of dorsal interneurons (Tozer et al., 2013), and also antagonizes SHH activity (Liem et al., 2000; Ulloa and Briscoe, 2007). However, prior to neural tube closure it is responsible for the promotion of non-neural ectodermal lineages (Stern, 2006; Ulloa and Briscoe, 2007). Given our differentiation protocol and timeline (i.e. differentiation factors applied from days 0 to 7), we hypothesized that introducing an opposing gradient of BMP4 at a very early time point would serve to inhibit neural differentiation and thus restrict MN formation.

We investigated several combinations of opposing PM and BMP4 gradients and found a universal response whereby BMP4 induced a significant spatial narrowing of the MN region (Fig. 3E), indicating that the differentiating cells were able to sense and correctly respond to the combined effects of the two signaling factors imposed within the microdevice. Furthermore, consistent with the known role of BMP4 and its maintenance of pluripotency (Zhang et al., 2010), we noted expression of the pluripotent ESC marker OCT4 (POU5F1 – Mouse Genome Informatics) (Fig. 3F), particularly in regions of high BMP4 concentrations and generally outside of the *HB9*^+^ permissive region. Combined, these results indicate that we are able to achieve a significant amount of control over the directed spatial differentiation of the ESCs with just two neural tube mediators.

### Transcription factor dynamics in controlled microenvironments

Morphogens such as SHH are generally thought to provide crude patterning cues that are later refined into sharp boundaries by transcription factor interactions present in the underlying gene regulatory network (GRN). Elucidating these transcription factor interactions is crucial for understanding the spatial and temporal development of the spinal cord. Currently, the dynamics of the neural tube GRN are poorly understood. *In vitro* experiments using bath applications of morphogens invariably elicits heterogeneous cellular responses making it difficult to parse out the underlying GRN. Exposing cells to physiological morphogen gradients allows users to tweak developmental parameters, such as morphogen concentration, gradient slope, temporal gradient changes etc., and monitor the downstream responses in a controlled and reproducible microenvironment.

**Fig. 4.**
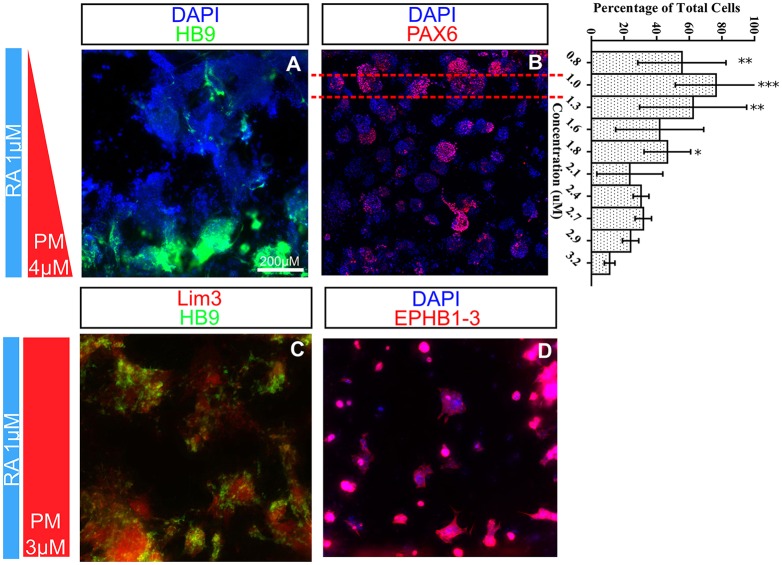
**Spatial and temporal expression of transcription factors *in vitro* consistent with *in vivo* expression.** (A) Staining of post-mitotic cells at day 6 uncovers the presence of non-*HB9*-expressing cells. (B) As MNs are developing in the ventral portion of the microdevice, PAX6 begins to regress dorsally (quantified as mean percent cells/bin ±s.d., *n*=3). Red dashed lines indicate example bin width. (C,D) After differentiation (day 9), the *HB9*^+^ MNs obtain a medial motor column identity (LIM3^+^) (C) and express markers of the hindbrain region (EPHB1-3^+^) (D). *P≤0.05, **P≤0.01, ***P≤0.001.

Indeed, each gradient experiment contains a wealth of biological data that can potentially be assessed using *in situ* immunostaining. We stained for several markers of the neural tube, including NKX2-2 and PAX6, to see if similar spatial patterning was occurring in the other DV domains. Despite repeated attempts to recapitulate the NKX2-2-positive V3 interneuron domain, no definitive patterning was evident even at day 6 when HB9^+^ MNs are present in the device. By contrast, at later time points (day 6) PAX6-positive cells began to display a subtle dorsal to ventral regression (Fig. 4B). This regression might be masked by the high concentration of RA used for the differentiation protocol, which is known to induce PAX6 expression (Novitch et al., 2003).

Given that the microdevice captures a single 100 µm dorsoventral slice of the neural tube somewhere on the anteroposterior (AP) axis (see Fig. 1A), we sought to confirm where on the AP axis this was. Because AP identity is not a known function of PM we applied a uniform concentration of both PM and RA, and assayed for markers of the AP axis. HB9^+^ MNs in the microdevice mostly expressed the AP markers EPHB1-3^+^, consistent with upper spinal cord or hindbrain identity (Fig. 4D), and did not express any markers of the thoracic or brachial spinal cord (Fig. S1). Furthermore, the MNs in the microdevice were mostly characteristic of those in the medial motor column (LIM3^+^; LHX3 – Mouse Genome Informatics) (Fig. 4C). Both of these observations correlate well with previously published work in standard culture systems (Gouti et al., 2014; Soundararajan, 2006).

### Novel temporal *in vitro* gradient studies in the microfluidic device

One aspect of neural development studies that is difficult to perform *ex vivo* is related to questions of temporal changes. Our device, coupled with fluorescent reporters, enables users to study real-time cell responses to imposed gradients. As an example, we used our fluorescent reporter cell line to address the question of whether cells in our microdevice were moving within the PM gradient, implying that PM acts as a chemoattractant, or if they were simply upregulating *HB9* in specific PM regions. In vertebrates, MN somas migrate laterally as they exit the cell cycle and settle in discrete clusters defined by their respective motor column (Price and Briscoe, 2004). However, in our device, time-lapse imaging of *HB9::*GFP cells during differentiation seems to suggest very little cell motility.

**Fig. 5.**
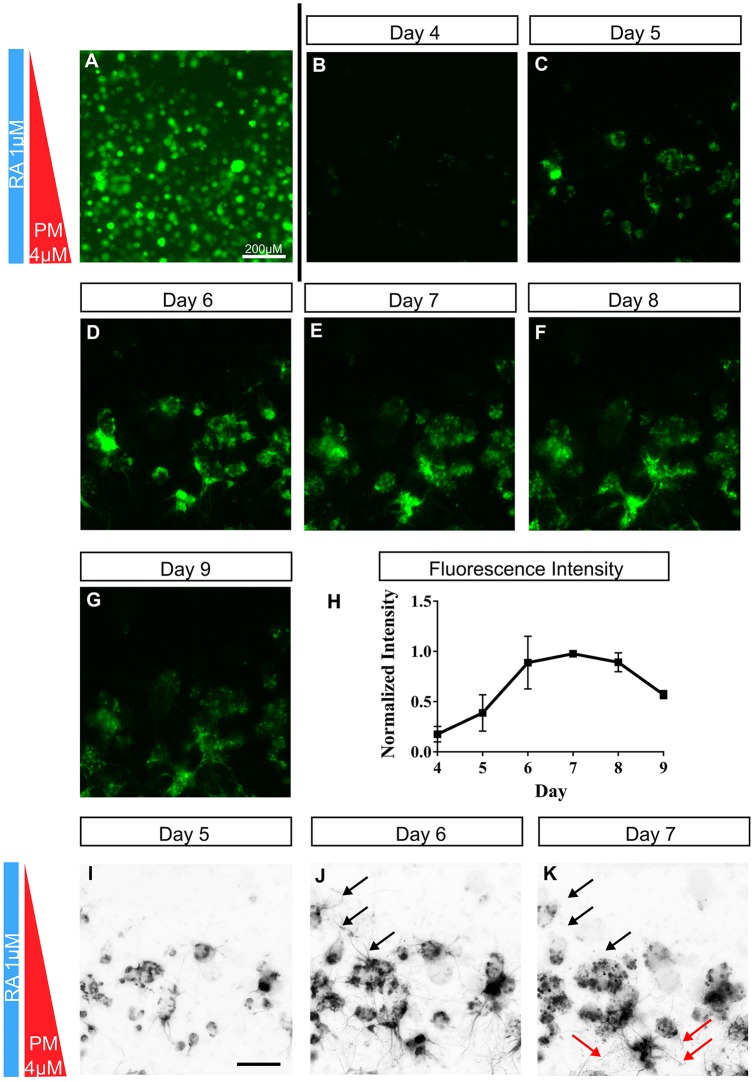
**Time-lapse imaging in the microdevice reveals dynamic expression patterns.** (A) Contrast has been artificially enhanced in this image to illustrate seeding density. (B-G) Images for subsequent days were acquired every 24 h at a constant intensity. (H) Quantification of GFP intensity over time reveals a pattern of expression similar to that found *in vivo* (mean intensity ±s.d., *n*=3)*.* (I-K) Inverted images are shown to enhance neurite visibility. Black arrows illustrate regions of omnidirectional neurite growth on day 6 that were redirected towards higher concentrations of PM (red arrows) on day 7.

Cells were seeded in a microdevice with a uniform density and imaged daily using a constant fluorescence intensity to assess cell motility (Fig. 5B-G). Fig. 5A shows the initial distribution of cells within the microdevice using enhanced contrast of intrinsic fluorescence. Notably, in all experiments cells tended to clump together despite their initial uniform distribution. This self-aggregation is similar to that seen in spheroids/organoids and is probably an intrinsic ESC process (Baillie-Johnson et al., 2015; van den Brink et al., 2014). Furthermore, within each cluster of differentiating cells there was a significant cellular rearrangement (Movie 1), which might represent translocations during the final stages of differentiation, similar to the medial-to-lateral shift seen in the neural tube. However, once differentiated, most cells are static (Fig. 5B-G; Movie 2). This strongly suggests a scenario in which cells respond to threshold stimulations of SHH and subsequently differentiate into neural subtypes within specific SHH concentration ranges. However, before forming HB9^+^ clusters, some single HB9^+^ cells do appear to have considerable motility with average velocities of 20-25 µm/h (Fig. S2; Movie 1). The reason for this behavior and the chemical fields driving motility are not known, but motile cells tend to move up the PM gradient (Fig. S2; Movie 1). Additionally, quantification of the fluorescence intensities in Fig. 5 reveals the temporal expression of *HB9*, which peaks at day 7 and begins to decline thereafter. This correlates well with *in vivo* data that suggests *HB9* is downregulated as MNs choose their specified motor column subtype (Davis-Dusenbery et al., 2014).

As a tangential note, another well-known gradient-induced response is axonal guidance (Sloan et al., 2015). Indeed, a common feature of all neural cells is the development and extension of neurites (Lodish et al., 2000). In the microdevice, MNs grown over 7 days exhibited a highly dynamic pattern of neurite extension and regression (Fig. 5I; Movie 2). Interestingly, the MN bundles appear to extend neurites omnidirectionally at day 6, and by day 7 some appear to have redirected their neurites up the PM gradient (Fig. 5J,K, arrows). The SHH pathway has, indeed, been implicated in axon guidance of dorsal interneurons that synapse with ventral neural populations and provide sensory and motor coordination (Charron et al., 2003). However, potential SHH-mediated guidance of MN projections has not been observed previously *in vitro* and warrants further investigation. Taken together, these data illustrate the ease of performing time-lapse studies using this platform, particularly when coupled with transgenic reporter stem cell lines.

### Generating overlapping orthogonal morphogen gradients

For experimental simplicity, each developmental patterning axis has historically been treated as separate entities. Although this provides a useful approximation to the conditions found in the neural tube, it potentially ignores important interactions between orthogonal and temporal signaling pathways. An interesting demonstration of this is the recent discovery that neural progenitor cells require a temporal pulse of WNT, a tail bud-derived signaling factor, in order to obtain a more posterior spinal cord identity (Gouti et al., 2014; Turner et al., 2014). The anteroposterior axis also plays a crucial role in delineating spinal motor columns, which are defined by their combinatorial Hox gene code established by opposing gradients of RA and WNT/FGF (Sockanathan et al., 2003). Thus, any biologically relevant *in vitro* platform for the recapitulation of spinal cord development would not be complete without integrating both primary axes as well as temporal perturbations.

**Fig. 6.**
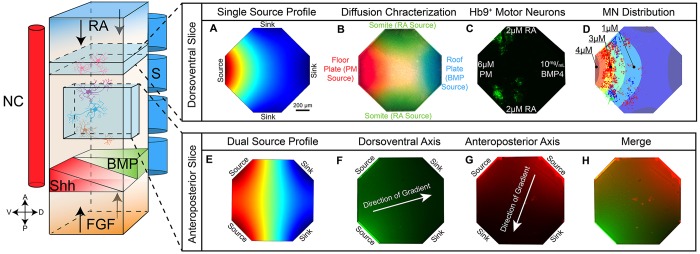
**Generating overlapping orthogonal gradients in a novel four-port microdevice.** Simply adding two additional diffusion ports provides an enhanced user platform with which one can recreate both an ‘Anteroposterior’ or ‘Dorsoventral’ slice of the neural tube. Introducing PM as a single source (equivalent to the neural tube floor plate) with flanking RA sources and an opposing BMP source induces a similar spatial pattern to that seen with the two-port microdevice. (A-D) Concentration profiles and demonstrated HB9 response for a dorsoventral slice of the notochord using four independent sources. (A) Simulated concentration profile for a single species using one source and three sinks. (B) Experimental demonstration of microdevice concentration profiles using each port as a single source for a colored dye. (C) Experimental HB9^+^ response to the indicated mediator gradients. (D) Color-coded HB9^+^ distribution plotted for three separate experiments. Calculated SHH concentration gradient is shown as background. (E-H) Concentration profiles demonstrated for an anteroposterior slice of the notochord using the four-channel device. (E) Computer simulation of concentration profiles using two adjacent inputs as a source. Profiles are approximately linear. (F,G) Experimental demonstration the linear gradient using the fluorescent dyes fluorescein (F) and rhodamine (G). (H) Superposition of linear gradients in F and G. NC, notochord; S, somite.

Given the successful and predicable differentiation of MNs in a simple two-channel microdevice, we sought to create mediator patterns that more faithfully explore *in vitro* spatiotemporal differentiations. To this end, we designed a four-channel microdevice to simulate opposing DV delivery of SHH (PM) and BMP4 as well as the orthogonal AP delivery of RA (Fig. 6). Fig. 6A shows the geometry of the microdevice along with a computer simulation demonstrating the gradient profile for a single source (PM in this case) with the other three ports serving as sinks. The isoconcentration lines for this source/sink configuration form arcs radiating outwards from the source. Application of other sources in the other channels will form similar arcs surrounding their respective sources, as shown experimentally in Fig. 6B where colored dyes are introduced into their respective ports. Dyes are intended to represent the developing *in vivo* gradients of PM (red), BMP4 (blue) and RA (green). Fig. 6C shows the differentiation of MNs that occurred under this diffusional landscape and Fig. 6D shows the distribution of HB9^+^ MNs as a function of the PM concentration. Subjecting *HB9::*GFP cells to opposing gradients of PM and BMP4 with flanking RA confirms previous two-channel experiments. However, unlike the previous experiments where MNs developed uniformly across the MN domain, here we see a bias towards the lateral edges of the microdevice (Fig. 6C,D). This pattern is also evident *in vivo* where terminally differentiated MNs settle in the lateral horn of the spinal cord (Jessell, 2000). The observation that this pattern is reproduced *in vitro* strongly suggests either a chemotactic effect of RA on the MN domain or an optimal concentration range of RA signaling. Both of these hypotheses warrant further investigation.

The four-channel microdevice can also be used to form other potentially biologically relevant concentration profiles depending solely on the loading of the respective channels. For example, the simple linear gradient used in the two-channel device is easily formed by loading adjacent channels with the same mediator in a dual-source configuration (Fig. 6E). The respective linear gradients are demonstrated experimentally for two fluorescent dyes, fluorescein and rhodamine (Fig. 6F,G) and their combined concentration pattern (Fig. 6H). Using different concentrations of fluorophores in each source or designing channels in the microdevice to enhance diffusion for a given set of channels can be used to rotate the respective concentration profiles relative to each other, generating an arbitrary angle of intersection between opposing gradients. This is clearly seen in Fig. 6G,H where the fluorophore concentration at each source is clearly different. Additional channels can also be easily added to increase the complexity of the concentration profiles and more closely mimic *in vivo* experiences (Smith et al., 2010).

## DISCUSSION

This article presents two novel microfluidic platforms capable of creating dynamic spatial and temporal microenvironments similar to those found during vertebrate neural development. As a demonstration of their utility, we have performed preliminary experiments that validate their ability to sustain stem cell growth, create tailored chemical microenvironments (particularly morphogen gradients), and recapitulate the primary aspects of neural tube organization. SHH, as the primary driver for the differentiation of ventral neural subtypes, directs MN differentiation within a fixed PM concentration range of roughly 3-4 µM, independent of the PM gradient. It should be noted that in our experiments a PM gradient is established, not a SHH gradient. It has been tacitly assumed throughout this article that activation of the SHH pathways through the agonist PM is equivalent to a SHH gradient. Additional investigation is warranted to validate this assumption. For all experiments, PM was used in conjunction with RA, a known inducer of neural differentiation (Bain et al., 1996). Experiments performed in the two-channel microdevice suggested that MN differentiation only occurred above a threshold RA concentration of 0.1 µM (data not shown) and was not a tangible function of RA gradients. However, in the four-channel microdevice with more extensive and realistic environments, the MNs appeared to prefer a particular combination of PM/RA signaling (Fig. 6C,D). Furthermore, a general anterior-to-posterior RA gradient does exist *in vivo* (del Corral and Storey, 2004) and could be investigated using the four-channel microdevice. Finally, we showed that the introduction of an opposing BMP4 gradient induces a more defined sharpening of the MN band through inhibition of neuroectodermal lineages (Stern, 2006).

This microfluidic platform represents a significant advancement over existing techniques, allowing the establishment of more biomimetic environments with which biologists will address complex developmental questions such as subtype specification mechanisms and interactions between signaling pathways, as well as transcription factor interactions. The current embodiment of the device, with its dual source/sink channels, as well as simple variants of it (Smith et al., 2010), allows the investigation of cellular differentiation in a user-defined concentration landscape. Furthermore, the successful operation of the device requires no more attention than standard cell culture processes.

Although *in vitro* MN differentiation and general neural development was the primary focus of this article, one can envisage therapeutic applications. For example, the ability to generate specific spatially defined populations of neural subtypes *in vitro* has enormous benefits in the remediation of nervous disorders such as amyotrophic lateral sclerosis (ALS) and spinal muscular atrophy, in which select neural subtypes preferentially die (Kanning et al., 2010). However, to recreate the early developmental events that result in the generation of distinct neural subtypes, we must first answer fundamental questions about the development of the neural tube that are not fully understood. Fortunately, this simple and powerful device provides the ability to build the neural tube environment from the ground up. Even with only three morphogens we are able to generate a single ‘slice’ of the neural tube (Fig. 1A; Fig. 6). Future work will concentrate on a combinatorial investigation using other chemical morphogens, including members of the FGF and TGFβ families, temporal effects as well as live-cell imaging of patterning events.

## MATERIALS AND METHODS

### Microdevice fabrication

The microdevice consists of a silicon chip with microfluidic channels and a cell culture chamber, bonded to a glass substrate. Double-side polished, silicon wafers (WRS Materials) were micromachined using standard photolithography and deep reactive ion etching (DRIE) to form microchannels on one side of the wafer (bottom) and an aligned cell chamber on the other side (top) with small (10 µm wide by 20 µm deep) connecting vias between the microchannels and the cell chamber, along two opposing edges. The silicon was anodically bonded to glass (Borofloat 33) on the bottom to seal the microchannels and provide a fluidic interconnect to the microdevice. Solution reservoirs were attached to the microfluidic channels using biomedical grade RTV adhesive (Factor II) to provide reagent flow to the microchannels. *HB9*::GFP cells in a Matrigel or Geltrix matrix were then introduced into the cell chamber and the chamber covered with a cut piece of standard microscope cover glass (0.17 mm thick). The entire device with filled solution reservoirs was then placed in an incubator at 37°C, 5% CO_2_ and allowed to culture. Flow through the microchannels was regulated using microfabricated, high resistance, on-chip fluidic restrictions in the fluidic microchannels. The small vias between the microchannels and the chamber permit flow-free diffusion of chosen mediators into the gel-filled cell culture chamber, by which concentration gradients can be imposed, much like those encountered in living tissue. COMSOL was utilized in designing the chip to optimize the channel geometry and achieve appropriate flow rates and concentration gradients. Microdevice characterization was performed using a 10 µM solution of fluorescein (Sigma) in water.

### MN differentiation

For microdevice experiments, transgenic *HB9::*GFP mouse embryonic stem cells (mESCs) (derived by the Cox Laboratory, The Jackson Laboratory) were plated onto mitomycin C-treated fibroblasts (The Jackson Laboratory) and allowed to grow for 2-3 days in ADFNK (Peljto et al., 2010; Wichterle et al., 2002) stem cell media (1:1 Neurobasal:Adv.-DMEM/F-12) containing leukemia inhibitory factor (EMD Millipore). Before plating onto the microdevice, fibroblasts were separated from the mESCs gravimetrically. Stem cells were then re-suspended in growth factor-reduced Geltrex (Life Technologies) at a concentration of 10^6^-10^7^ cells/ml and plated onto the microdevice chamber. A cover slip was applied over the chamber and the cover slip edges were sealed with biomedical grade silicone to prevent potential evaporation. The media reservoirs were loaded with appropriate concentrations of purmorphamine (SHH agonist, Cayman Chemical), retinoic acid (Sigma) and BMP4 (R&D Systems) in ADFNK media. Cell-loaded microdevices were then incubated at 37°C under 5% CO_2_ ambient with microchannel fluid flow to both perfuse cells and establish mediator gradients. Solution reservoirs were refreshed with media as needed, usually daily. Flow rates for all devices were ∼100 µl/h throughout the duration of the experiment. This flow was more than sufficient to maintain a stable concentration gradient across the cell chamber and adequately perfuse the cells. Although fluid flow existed in the fluidic microchannels, flow across the cells was essentially zero except in the first few minutes after initial introduction of reagents, producing an environment closely mimicking living tissue. Cells were maintained in the microdevice for up to 9 days without noticeable loss of vitality. After the designated experiment timeline (typically 7 days) cells were fixed *in situ* with 4% paraformaldehyde for twenty minutes. For immunocytochemistry, PAX6 [1:1000; deposited to the Developmental Studies Hybridoma Bank (DSHB) by A. Kawakami (DSHB Hybridoma Product PAX6)], LIM3 [1:10; deposited to the DSHB by T. M. Jessell and S. Brenner-Morton (DSHB Hybridoma Product 67.4E12)] and Eph receptors B1-3 [1:10; deposited to the DSHB by Z. Kaprielian (DSHB Hybridoma Product mAb EfB1-3)] antibodies were all incubated for 24 h at 4°C. Devices were washed and rhodamine anti-mouse secondary antibodies (1:100; Jackson ImmunoResearch) were then applied to the devices and incubated for 24 h at 4°C.

### Data analysis

Imaging was performed on a Zeiss AxioObserver Z1 at 50× magnification and exposure times calibrated for constant intensity. Images of HB9^+^/GFP cells were thresholded and counted using ImageJ (NIH) using ten 100-µm-wide bins that spanned the width of the device (1 mm). At the low magnifications used here (50×), individual cells were not resolved and fluorescent intensities were used to quantify cells. An ‘unsharp mask’, which provides enhanced contrast at the expense of additional noise, was used. Data were normalized to account for differences in cell density between devices by dividing the HB9^+^ cell count per bin by the total number of HB9^+^ cells in the device. Statistically significant bins were analyzed using a two-way ANOVA against the assumption of uniform distribution of cells (i.e. 10% of the cells in each bin). At least three replicates were used for each experiment.

### COMSOL modeling

COMSOL ‘Transport of Diluted Species’ physics tree was used for all simulations. Simulation geometry was based on actual device geometry. All geometric entities were assigned ‘Water’ as a material. The viscosity of the ‘Water’ in the cell chamber was defined as 100× the default viscosity, which approximates the viscosity of Matrigel. In the ‘Transport of Diluted Species’ tree, we defined two additional ‘Transport Properties’ domains, which are used to define the velocity of the source and sink channels, respectively. The velocities were given as Vsink and Vsource as a ‘User defined’ velocity field in the positive *x* direction. Velocities used for the simulation corresponded to fluid flow rates of ∼100 µl/h. Two ‘Inflow’ boundary conditions were used, which define the 1 and 0 concentrations of the source and sink, respectively, as well as an ‘Outflow’ boundary condition for the fluid leaving the system. A default ‘Normal’ mesh was used to mesh the simulation geometry. Lastly, a ‘Time-dependent’ study step was used for all calculations to obtain diffusion dynamics.

## References

[DEV126847C1] Amadi O. C., Steinhauser M. L., Nishi Y., Chung S., Kamm R. D., McMahon A. P. and Lee R. T. (2010). A low resistance microfluidic system for the creation of stable concentration gradients in a defined 3D microenvironment. *Biomed. Microdevices* 12, 1027-1041. 10.1007/s10544-010-9457-7 20661647 PMC3119200

[DEV126847C2] Arber S., Han B., Mendelsohn M., Smith M., Jessell T. M. and Sockanathan S. (1999). Requirement for the homeobox gene Hb9 in the consolidation of motor neuron identity. *Neuron* 23, 659-674. 10.1016/S0896-6273(01)80026-X 10482234

[DEV126847C3] Baillie-Johnson P., van den Brink S. C., Balayo T., Turner D. A. and Martinez Arias A. (2015). Generation of aggregates of mouse embryonic stem cells that show symmetry breaking, polarization and emergent collective behaviour in vitro. *J. Vis. Exp.*, e53252 10.3791/53252 PMC4692741 26650833

[DEV126847C4] Bain G., Ray W. J., Yao M. and Gottlieb D. I. (1996). Retinoic acid promotes neural and represses mesodermal gene expression in mouse embryonic stem cells in culture. *Biochem. Biophys. Res. Commun.* 223, 691-694. 10.1006/bbrc.1996.0957 8687458

[DEV126847C5] Bushati N. and Briscoe J. (1994). Regulation of neuronal subtype identity in the vertebrate neural tube (neuronal subtype identity regulation). *eLS*.

[DEV126847C6] Chamberlain C. E., Jeong J., Guo C., Allen B. L. and McMahon A. P. (2008). Notochord-derived Shh concentrates in close association with the apically positioned basal body in neural target cells and forms a dynamic gradient during neural patterning. *Development* 135, 1097-1106. 10.1242/dev.013086 18272593

[DEV126847C7] Charron F., Stein E., Jeong J., McMahon A. P. and Tessier-Lavigne M. (2003). The morphogen sonic hedgehog is an axonal chemoattractant that collaborates with netrin-1 in midline axon guidance. *Cell* 113, 11-23. 10.1016/S0092-8674(03)00199-5 12679031

[DEV126847C8] Davis-Dusenbery B. N., Williams L. A., Klim J. R. and Eggan K. (2014). How to make spinal motor neurons. *Development* 141, 491-501. 10.1242/dev.097410 24449832

[DEV126847C9] del Corral R. D. and Storey K. G. (2004). Opposing FGF and retinoid pathways: a signalling switch that controls differentiation and patterning onset in the extending vertebrate body axis. *Bioessays* 26, 857-869. 10.1002/bies.20080 15273988

[DEV126847C10] Dessaud E., McMahon A. P. and Briscoe J. (2008). Pattern formation in the vertebrate neural tube: a sonic hedgehog morphogen-regulated transcriptional network. *Development* 135, 2489-2503. 10.1242/dev.009324 18621990

[DEV126847C11] Dessaud E., Ribes V., Balaskas N., Yang L. L., Pierani A., Kicheva A., Novitch B. G., Briscoe J. and Sasai N. (2010). Dynamic assignment and maintenance of positional identity in the ventral neural tube by the morphogen sonic hedgehog. *PLoS Biol.* 8, e1000382 10.1371/journal.pbio.1000382 20532235 PMC2879390

[DEV126847C12] Diez del Corral R., Olivera-Martinez I., Goriely A., Gale E., Maden M. and Storey K. (2003). Opposing FGF and retinoid pathways control ventral neural pattern, neuronal differentiation, and segmentation during body axis extension. *Neuron* 40, 65-79. 10.1016/S0896-6273(03)00565-8 14527434

[DEV126847C13] Dodson C. S., Peresypkin A. V., Rengarajan K., Wu S. and Nickerson J. M. (2002). Diffusion coefficients of retinoids. *Curr. Eye Res.* 24, 66-74. 10.1076/ceyr.24.1.66.5428 12187497

[DEV126847C14] Ericson J., Briscoe J., Rashbass P., van Heyningen V. and Jessell T. M. (1997). Graded sonic hedgehog signaling and the specification of cell fate in the ventral neural tube. *Cold Spring Harb. Symp. Quant. Biol.* 62, 451-466. 9598380

[DEV126847C15] Gouti M., Tsakiridis A., Wymeersch F. J., Huang Y., Kleinjung J., Wilson V. and Briscoe J. (2014). In vitro generation of neuromesodermal progenitors reveals distinct roles for wnt signalling in the specification of spinal cord and paraxial mesoderm identity. *PLoS Biol.* 12, e1001937 10.1371/journal.pbio.1001937 25157815 PMC4144800

[DEV126847C16] Jessell T. M. (2000). Neuronal specification in the spinal cord: inductive signals and transcriptional codes. *Nat. Rev. Genet.* 1, 20-29. 10.1038/35049541 11262869

[DEV126847C17] Kanning K. C., Kaplan A. and Henderson C. E. (2010). Motor neuron diversity in development and disease. *Annu. Rev. Neurosci.* 33, 409-440. 10.1146/annurev.neuro.051508.135722 20367447

[DEV126847C18] Le Dréau G., Garcia-Campmany L., Rabadán M. A., Ferronha T., Tozer S., Briscoe J. and Marti E. (2012). Canonical BMP7 activity is required for the generation of discrete neuronal populations in the dorsal spinal cord. *Development* 139, 259-268. 10.1242/dev.074948 22159578 PMC3243093

[DEV126847C19] Liem K. F., Jessell T. M. and Briscoe J. (2000). Regulation of the neural patterning activity of sonic hedgehog by secreted BMP inhibitors expressed by notochord and somites. *Development* 127, 4855-4866. 11044400 10.1242/dev.127.22.4855

[DEV126847C20] Liu J.-P., Laufer E. and Jessell T. M. (2001). Assigning the positional identity of spinal motor neurons: rostrocaudal patterning of Hox-c expression by FGFs, Gdf11, and retinoids. *Neuron* 32, 997-1012. 10.1016/S0896-6273(01)00544-X 11754833

[DEV126847C21] Lodish H., Berk, A., Zipursky S. L. Matsudaira P., Baltimore D. and Darnell J. (2000). Molecular cell biology, 4th edn. New York: W. H. Freeman.

[DEV126847C22] Łopacińska J. M., Emnéus J. and Dufva M. (2013). Poly(Dimethylsiloxane) (PDMS) affects gene expression in PC12 cells differentiating into neuronal-like cells. *PLoS ONE* 8, e53107 10.1371/journal.pone.0053107 23301028 PMC3536795

[DEV126847C23] Novitch B. G., Wichterle H., Jessell T. M. and Sockanathan S. (2003). A requirement for retinoic acid-mediated transcriptional activation in ventral neural patterning and motor neuron specification. *Neuron* 40, 81-95. 10.1016/j.neuron.2003.08.006 14527435

[DEV126847C24] Park J. Y., Kim S.-K., Woo D.-H., Lee E.-J., Kim J.-H. and Lee S.-H. (2009). Differentiation of neural progenitor cells in a microfluidic chip-generated cytokine gradient. *Stem Cells* 27, 2646-2654. 10.1002/stem.202 19711444

[DEV126847C25] Peljto M., Dasen J. S., Mazzoni E. O., Jessell T. M. and Wichterle H. (2010). Functional diversity of ESC-derived motor neuron subtypes revealed through intraspinal transplantation. *Cell Stem Cell* 7, 355-366. 10.1016/j.stem.2010.07.013 20804971 PMC2933095

[DEV126847C26] Price S. R. and Briscoe J. (2004). The generation and diversification of spinal motor neurons: signals and responses. *Mech. Dev.* 121, 1103-1115. 10.1016/j.mod.2004.04.019 15296975

[DEV126847C27] Regehr K. J., Domenech M., Koepsel J. T., Carver K. C., Ellison-Zelski S. J., Murphy W. L., Schuler L. A., Alarid E. T. and Beebe D. J. (2009). Biological implications of polydimethylsiloxane-based microfluidic cell culture. *Lab Chip* 9, 2132-2139. 10.1039/b903043c 19606288 PMC2792742

[DEV126847C28] Ribes V. and Briscoe J. (2009). Establishing and interpreting graded Sonic Hedgehog signaling during vertebrate neural tube patterning: the role of negative feedback. *Cold Spring Harb. Perspect. Biol.* 1, a002014 10.1101/cshperspect.a002014 20066087 PMC2742090

[DEV126847C29] Roelink H., Porter J. A., Chiang C., Tanabe Y., Chang D. T., Beachy P. A. and Jessell T. M. (1995). Floor plate and motor neuron induction by different concentrations of the amino-terminal cleavage product of sonic hedgehog autoproteolysis. *Cell* 81, 445-455. 10.1016/0092-8674(95)90397-6 7736596

[DEV126847C30] Shin Y., Kim H., Han S., Won J., Jeong H. E., Lee E.-S., Kamm R. D., Kim J.-H. and Chung S. (2013). Extracellular matrix heterogeneity regulates three-dimensional morphologies of breast adenocarcinoma cell invasion. *Adv. Healthc. Mater.* 2, 790-794. 10.1002/adhm.201200320 23184641 PMC4074172

[DEV126847C31] Sinha S. and Chen J. K. (2006). Purmorphamine activates the Hedgehog pathway by targeting Smoothened. *Nat. Chem. Biol.* 2, 29-30. 10.1038/nchembio753 16408088

[DEV126847C32] Sloan T. F. W., Qasaimeh M. A., Juncker D., Yam P. T. and Charron F. (2015). Integration of shallow gradients of Shh and Netrin-1 guides commissural axons. *PLoS Biol.* 13, e1002119 10.1371/journal.pbio.1002119 25826604 PMC4380419

[DEV126847C33] Smith J. L. and Schoenwolf G. C. (1997). Neurulation: coming to closure. *Trends Neurosci.* 20, 510-517. 10.1016/S0166-2236(97)01121-1 9364665

[DEV126847C34] Smith R. L., Demers C. J. and Collins S. D. (2010). Microfluidic device for the combinatorial application and maintenance of dynamically imposed diffusional gradients. *Microfluid. Nanofluidics* 9, 613-622. 10.1007/s10404-010-0574-7

[DEV126847C35] Sockanathan S., Perlmann T. and Jessell T. M. (2003). Retinoid receptor signaling in postmitotic motor neurons regulates rostrocaudal positional identity and axonal projection pattern. *Neuron* 40, 97-111. 10.1016/S0896-6273(03)00532-4 14527436

[DEV126847C36] Soundararajan P. (2006). Motoneurons derived from embryonic stem cells express transcription factors and develop phenotypes characteristic of medial motor column neurons. *J. Neurosci.* 26, 3256-3268. 10.1523/JNEUROSCI.5537-05.2006 16554476 PMC6674087

[DEV126847C37] Stern C. D. (2006). Neural induction: 10 years on since the ‘default model’. *Curr. Opin. Cell Biol.* 18, 692-697. 10.1016/j.ceb.2006.09.002 17045790

[DEV126847C38] Tozer S., Le Dréau G., Marti E. and Briscoe J. (2013). Temporal control of BMP signalling determines neuronal subtype identity in the dorsal neural tube. *Development* 140, 1467-1474. 10.1242/dev.090118 23462473 PMC3596990

[DEV126847C39] Turner D. A., Hayward P. C., Baillie-Johnson P., Rué P., Broome R., Faunes F. and Martinez Arias A. (2014). Wnt/β-catenin and FGF signalling direct the specification and maintenance of a neuromesodermal axial progenitor in ensembles of mouse embryonic stem cells. *Development* 141, 4243-4253. 10.1242/dev.112979 25371361 PMC4302903

[DEV126847C40] Ulloa F. and Briscoe J. (2007). Morphogens and the control of cell proliferation and patterning in the spinal cord. *Cell Cycle* 6, 2640-2649. 10.4161/cc.6.21.4822 17912034

[DEV126847C41] van den Brink S. C., Baillie-Johnson P., Balayo T., Hadjantonakis A.-K., Nowotschin S., Turner D. A. and Martinez Arias A. (2014). Symmetry breaking, germ layer specification and axial organisation in aggregates of mouse embryonic stem cells. *Development* 141, 4231-4242. 10.1242/dev.113001 25371360 PMC4302915

[DEV126847C42] Wichterle H., Lieberam I., Porter J. A. and Jessell T. M. (2002). Directed differentiation of embryonic stem cells into motor neurons. *Cell* 110, 385-397. 10.1016/S0092-8674(02)00835-8 12176325

[DEV126847C43] Wilson L. and Maden M. (2005). The mechanisms of dorsoventral patterning in the vertebrate neural tube. *Dev. Biol.* 282, 1-13. 10.1016/j.ydbio.2005.02.027 15936325

[DEV126847C44] Zhang K., Li L., Huang C., Shen C., Tan F., Xia C., Liu P., Rossant J. and Jing N. (2010). Distinct functions of BMP4 during different stages of mouse ES cell neural commitment. *Development* 137, 2095-2105. 10.1242/dev.049494 20504958

